# Patients’ Experiences of a National Patient Portal and Its Usability: Cross-Sectional Survey Study

**DOI:** 10.2196/45974

**Published:** 2023-06-30

**Authors:** Saija Simola, Iiris Hörhammer, Yuhui Xu, Annika Bärkås, Asbjørn Johansen Fagerlund, Josefin Hagström, Mari Holmroos, Maria Hägglund, Monika Alise Johansen, Bridget Kane, Anna Kharko, Isabella Scandurra, Sari Kujala

**Affiliations:** 1 Department of Computer Science Aalto University Espoo Finland; 2 Department of Women’s and Children’s Health Uppsala University Uppsala Sweden; 3 Norwegian Centre for E-health Research University Hospital of North Norway Tromsø Norway; 4 Kela The Social Insurance Institution of Finland Helsinki Finland; 5 Karlstad University Business School Karlstad Sweden; 6 Faculty of Health University of Plymouth Plymouth United Kingdom; 7 Center for Empirical Research on Information Systems Örebro University Örebro Sweden

**Keywords:** patient portal, perceived usability, System Usability Scale, electronic health record, patient experiences, patient-accessible electronic health records, national survey

## Abstract

**Background:**

Patient portals not only provide patients with access to electronic health records (EHRs) and other digital health services, such as prescription renewals, but they can also improve patients’ self-management, engagement with health care professionals (HCPs), and care processes. However, these benefits depend on patients’ willingness to use patient portals and, ultimately, their experiences with the usefulness and ease of use of the portals.

**Objective:**

This study aimed to investigate the perceived usability of a national patient portal and the relationship of patients’ very positive and very negative experiences with perceived usability. The study was aimed to be the first step in developing an approach for benchmarking the usability of patient portals in different countries.

**Methods:**

Data were collected through a web-based survey of the My Kanta patient portal’s logged-in patient users in Finland from January 24, 2022, to February 14, 2022. Respondents were asked to rate the usability of the patient portal, and the ratings were used to calculate approximations of the System Usability Scale (SUS) score. Open-ended questions asked the patients about their positive and negative experiences with the patient portal. The statistical analysis included multivariate regression, and the experience narratives were analyzed using inductive content analysis.

**Results:**

Of the 1,262,708 logged-in patient users, 4719 responded to the survey, giving a response rate of 0.37%. The patient portal’s usability was rated as good, with a mean SUS score of 74.3 (SD 14.0). Reporting a very positive experience with the portal was positively associated with perceived usability (β=.51; *P*<.001), whereas reporting a very negative experience was negatively associated with perceived usability (β=−1.28; *P*<.001). These variables explained 23% of the variation in perceived usability. The information provided and a lack of information were the most common positive and negative experiences. Furthermore, specific functionalities, such as prescription renewal and the ease of using the patient portal, were often mentioned as very positive experiences. The patients also mentioned negative emotions, such as anger and frustration, as part of their very negative experiences.

**Conclusions:**

The study offers empirical evidence about the significant role of individual experiences when patients are evaluating the usability of patient portals. The results suggest that positive and negative experiences provide relevant information that can be used for improving the patient portal’s usability. Usability should be improved so that patients receive information efficiently, easily, and quickly. Respondents would also appreciate interactive features in the patient portal.

## Introduction

### Background

Interest in more extensive use of eHealth services, such as patient portals, has increased in many countries. Sun et al [1] define patient portals as “web-based platforms for patients’ access to their health information from a health organization’s electronic health record (EHR).” Hagström et al [2] explain that an EHR contains clinical information such as notes from appointments, medication information, and diagnostic information.

Patient portals and patients’ access to their EHRs via patient portals have been shown to improve patient self-management [3-6], enhance patient understanding of health information [5], foster patient safety [4], and improve a patient’s relationship with health care professionals (HCPs) [3-8] and their engagement in care processes [9]. Establishing and providing access to patients’ EHRs is an integral part of people-centered health systems in the World Health Organization’s global strategy for digital health [10]. In a recent study, Kinney and Sankaranarayanan [11] suggested that the use of patient portals may improve patients’ perceptions of their health care and the health care system.

To reap the benefits of patient portals, patients must be willing to use them. Usability is an important factor supporting patient engagement with a patient portal [12]. The ISO-9241-11 standard defines usability as the *“*extent to which a product can be used by specified users to achieve specified goals with effectiveness, efficiency and satisfaction in a specified context of use*”* [13]. In addition, usability problems can create negative emotions for users [14,15], whereas perceived good usability may lead to positive emotions [15].

Patient portals have faced various types of usability challenges. Previous research in the United States, Sweden, Norway, and Finland identified usability problems with patient portals, such as difficulties with navigation [16-18], assumptions of nonexistent functionalities [17], difficulties with interpretation of health information [6,16,18,19] and test results [20-22], and concerns about data privacy and security [23,24]. Having missing or incorrect notes [19,22] has also been found to be a cause of negative experiences.

The System Usability Scale (SUS) is a questionnaire used to assess usability [25], and it is the most commonly used when developing eHealth services [26,27]. Although it is possible to compare usability using the SUS [27,28], it does not enable us to pinpoint specific reasons for poor usability [29]. Therefore, in this study, we explored whether qualitative analysis of patients’ very positive and very negative experiences could provide information on how the usability of the patient portal could be improved.

### My Kanta Patient Portal

My Kanta is a Finnish national patient portal that provides access to patients’ health data [30]. My Kanta was established in 2010, and the annual number of registered persons rose to 3.143 million between 2010 and 2019 [31]. In 2021, about 92% of adults used My Kanta [19]. My Kanta provides EHR access, including patient records (eg, from primary and specialist care), diagnoses, medical information, prescriptions and renewal, organ donation testament and living will, European Union (EU) digital COVID-19 vaccination certificate, laboratory test results, radiology results, and medical certificates [32]. The portal allows adult users to act on behalf of an adult (with consent) or a child [33]. Patients can permit or restrict access to their EHR by HCPs from other health care providers [34]. In addition, HCPs can set a standard delay for showing data in My Kanta, and the data will be shown after the HCP has finalized them [35]. Booking appointments and real-time contact between patients and HCPs are not available [33].

In previous studies, patients’ experiences with the My Kanta patient portal have been positive. Kainiemi et al [36] found that patients were satisfied with My Kanta, whereas pharmacy customers felt that My Kanta was clear and easy to use, and they used it mostly for viewing prescriptions [37]. Viewing and renewing prescriptions are some of the most commonly used features of My Kanta [19,23,38] and are perceived as some of its most useful features [19]. Older adults reported that My Kanta was easy to use, although security and privacy issues were common concerns [38], and they used it to manage their health information or because of the patient portal’s impact on their health behavior [38].

### Aims and Research Questions

The study investigated patients’ positive and negative experiences with the My Kanta patient portal and how these experiences related to the perceived usability of the portal. There is preliminary evidence that individual positive and negative experiences are associated with a product’s overall evaluation [14]. This study approached usability with a survey combining qualitative and quantitative questions to identify the usability problems and benefits of using the patient portal.

The study also aimed to be the first step in developing an approach for benchmarking the usability of patient portals in different countries. As improving usability of EHR systems has continued to be challenging over the years [39], it is important to be able to compare the usability aspects of national systems and identify their strengths and weaknesses.

Our research questions were as follows:

1. Are patients’ reported very positive and very negative experiences of using a patient portal related to its perceived usability?
2. What kinds of very positive and very negative experiences of a patient portal do patients have?

## Methods

A web-based survey was created to investigate patients’ experiences with a national patient portal, My Kanta following the CHERRIES (Checklist for Reporting Results of Internet E-Surveys) [40] (Multimedia Appendix 1).

### Survey

The web-based survey was part of the NORDeHEALTH project [41] in Finland, Estonia, Norway, and Sweden. The methodology of the NORDeHEALTH 2022 Patient Survey is fully described elsewhere [42]. Researchers from all 4 countries participated in the survey design, and the study was performed at the same time in all 4 countries. The survey was tested beforehand with 4 volunteer participants.

This study focused on the results of the usability questions in Finland. The questionnaire consisted of Likert-scale, multiple-choice, and open-ended questions (Multimedia Appendix 2). The survey was performed using Webropol (version 3.0). The quantitative variables were usability measured with a 3-item version of the usability metric for user experience (UMUX) instrument [43], reporting a very positive experience, reporting a very negative experience, age, gender, subjective health status, education, having received care in the past 24 months, and having an HCP education. The web-based questionnaire was dynamic; only respondents who reported having a very positive or very negative experience with the patient portal were asked to describe this experience in the open-ended follow-up question.

### Data Collection

The responses were collected with a web-based survey of logged-in patient users between January 24, 2022, and February 14, 2022. An invitation to voluntarily participate in the study, which included a survey link and study information, was shown to patients at the time point of logging out from the patient portal. The participants could respond to the survey in either Finnish or Swedish, Finland’s 2 official languages.

### Statistical Analysis

Following Lewis et al [44], we calculated the SUS score from 2 of the UMUX items using a corrective regression formula. The Cronbach α value for usability was .73, indicating acceptable internal consistency, especially for a short scale with only 3 items [45].

We fitted multivariate regression models to investigate the association of key independent variables with perceived usability and the robustness of these associations to relevant confounders. All statistical analyses were performed using Stata (version 17.0; StataCorp LLC).

Univariate analysis was performed for each independent variable to examine its association with the dependent variable. In addition to the key binary variables (reporting a very positive experience or reporting a very negative experience), the analysis included the following relevant confounders: age, gender, subjective health status, education, having received care in the past 24 months, and having an HCP education. The variables with a statistically significant association with the dependent variable in the univariate analyses were included in the multivariate analysis.

In the multivariate modeling, we first fitted a model (A) to test the associations between having reported a very positive experience and having reported a very negative experience with the dependent variable usability. In the second model (B), adjustments for subjective health status and health care use were added. In the third model (C), the sociodemographic factors of age, gender, and education level were applied. *P*<.05 was considered significant in all analyses. The variance inflation factors for the independent variables were calculated to test multicollinearity. These were all <1.06, indicating that multicollinearity was not a concern in this study [46].

### Qualitative Analysis

Qualitative analyses were conducted using ATLAS.ti software (version 22.2.5.0; ATLAS.ti Scientific Software Development GmbH) and Excel (2022; Microsoft Corp). Positive and negative experiences were analyzed separately. In addition, expressions of emotion were gathered and coded. To facilitate the detection of similarities in the responses, individual responses were organized in alphabetical order. The responses were then analyzed using inductive content analysis inspired by Bengtsson [47] and Harahap et al [48]. A participant’s response might have >1 code if it included several topics. Code was generated using in vivo coding.

A total of 92 preliminary codes from the codebook of very positive experiences were combined into 35 main themes and 8 final themes. For the very negative experiences, 161 preliminary codes were combined into 43 main themes and 9 final themes. The first author performed the analyses and the preliminary coding. Another author helped to analyze unclear answers. Two authors verified the codes used and the final themes.

### Ethics Approval, Informed Consent, and Participation

The study protocol was reviewed and approved by the Aalto University research ethics committee (D/957/03.04/2020 Z). Participation in the study was voluntary, and no incentives were offered. The survey was anonymous. The survey invitation included study information and the privacy notice, and answering the questionnaire was regarded as informed consent to participate. The informed consent allows secondary analysis without additional consent.

## Results

### Patient Characteristics

Of the 1,262,708 logged-in My Kanta patient users, 4719 responded to the survey, giving a response rate of 0.37%. Table 1 reports on the descriptive statistics of the respondents. The respondents were more often women (3422/4719, 72.52%) than men (1224/4719, 25.94%), and the women had a higher education degree (2828/4719, 59.93%). The respondents were older than My Kanta users in general. During the survey collection period, patient users aged ≥51 years made up 44% of the unique users of My Kanta. The proportion of the respondents who were HCPs (1026/4719, 21.74%) was larger than that of the HCPs among Finland’s population: in 2020, HCPs and social care professionals made up approximately 12.5% of the working-age population (aged 15-64 years) [49].

**Table 1:** Respondent characteristics (n=4719).

Characteristic			Respondents, n (%)
**Age range (years)**			
	15-24	86 (1.82)	
	25-34	224 (4.75)	
	35-44	361 (7.65)	
	45-54	595 (12.61)	
	55-64	1160 (24.58)	
	65-74	1596 (33.82)	
	75-84	620 (13.14)	
	≥85	52 (1.1)	
	Not reported	25 (0.53)	
**Gender**			
	Woman	3422 (72.52)	
	Man	1224 (25.94)	
	Nonbinary or not reported	73 (1.55)	
**Education**			
	No formal education	19 (0.40)	
	Elementary education	474 (10.04)	
	Upper secondary education	1217 (25.79)	
	Further vocational education	1033 (21.89)	
	Higher education ≤3 years (Bachelor’s degree)	826 (17.5)	
	Higher education >3 years (Master’s degree)	886 (18.78)	
	Doctoral or other higher education	83 (1.76)	
	Other or not reported	181 (3.84)	
**HCP^a^ education**			
	Yes	1026 (21.74)	
	No	3586 (75.99)	
	Not reported	107 (2.27)	

^a^HCP: health care professional.

### Association of Patient Characteristics and Having Reported Positive and Negative Experiences With Overall Perceived Usability

The mean SUS score was 74.3 (SD 14.0), indicating a good level of usability [50]. The univariate analysis showed that the independent variables of reporting a very positive experience (*P*<.001), reporting a very negative experience (*P*<.001), having received care during the past 24 months (*P*=.001), subjective health (*P*<.001), gender (*P*=.002), age (*P*<.001), and education (*P*<.001) were significantly associated with usability and therefore included in the multivariate analysis. Having an HCP education was not statistically significantly associated (*P*=.62) with usability and was therefore dropped from the multivariate analysis.

In the multivariate analysis (Table 2), reporting a very positive experience with the My Kanta patient portal was positively associated with perceived usability (β=.51; *P*<.001), whereas reporting a very negative experience was negatively associated with perceived usability (β=−1.28; *P*<.001). These variables explain 23% of the variation in perceived usability. Both associations remained when controlling for recent health care use and subjective health status (model B) and sociodemographic factors (model C; Table 2). Better subjective health status, older age, gender: man, and lower education were associated with higher subjective usability (model C; Table 2).

**Table 2:** Associations of having reported very positive and very negative experiences with subjective usability.

	Model A^a^		Model B^a^		Model C^a^	
	ß (SE)	*P* value	ß (SE)	*P* value	ß (SE)	*P* value
Reporting very positive experience (reference: no)	.51 (0.03)	<.001	.51 (0.03)	<.001	.49 (0.03)	<.001
Reporting very negative experience (reference: no)	−1.28 (0.04)	<.001	−1.27 (0.04)	<.001	−1.20 (0.04)	<.001
Received care in past 24 months (reference: no)	N/A^b^	N/A	.32 (0.09)	<.001	.29 (0.09)	.002
Subjective health status	N/A	N/A	.07 (0.02)	.001	.10 (0.02)	<.001
Age	N/A	N/A	N/A	N/A	.03 (0.01)	.003
Gender (reference: woman)	N/A	N/A	N/A	N/A	.09 (0.04)	.03
Education	N/A	N/A	N/A	N/A	−.06 (0.01)	<.001

^a^*R*^2^=0.23.

^b^N/A: not applicable.

### Frequency of Patients’ Very Positive and Very Negative Experiences

Of the 4719 respondents, 1837 (38.93%) answered the open-ended question about their very positive experiences, whereas 1305 (27.65%) reported very negative experiences. The number of words per a very positive experience narrative varied from 1 to 222 (mean 9.0, SD 10.9) and very negative experience narratives included from 1 to 478 (mean 22.0, SD 27.9) words. Although there were fewer very negative experiences than very positive experiences, the responses relating to very negative experiences were typically longer than those relating to very positive experiences.

### Patients’ Positive Experiences

More than half (939/1837, 51.12%) of the respondents mentioned that the patient portal provides relevant information. The respondents most often valued HCPs’ notes and other information, such as blood test and examination results as well as prescriptions (Table 3).

**Table 3:** Perceived very positive experiences (n=1837).

Themes		Values, n (%)^a^
**Provides information about...**		
	Health care professional notes	472 (25.69)
	Tests and examinations	335 (18.24)
	Prescriptions	239 (13.01)
	Terms of prescriptions	68 (3.7)
	Diagnoses	41 (2.23)
	Information available about myself	28 (1.52)
	Vaccinations	20 (1.09)
**Specific functionality**		
	Renewing prescriptions	566 (30.81)
	EU^b^ digital COVID-19 vaccination certificate	184 (10.02)
	Printing or uploading	65 (3.54)
	Acting on behalf of adult or child	25 (1.36)
	Living will or organ donation testament	20 (1.09)
	SMS text message notifications of prescriptions	9 (0.49)
	Consent to, or denial of, sharing own data	5 (0.27)
**General quality of the patient portal**		
	Easy	462 (25.15)
	Quick process	72 (3.92)
	Organized	49 (2.67)
	Quick	20 (1.09)
	System itself	20 (1.09)
	Reliable	6 (0.33)
	Secure	5 (0.27)
**Convenience of patient portal access**		
	Easy and fast to find and access information	368 (20.03)
	No need to call or contact health care services	56 (3.05)
	Available without depending on time or place	41 (2.23)
	Can be checked at leisure	23 (1.25)
**Supports recall**		
	Care history	137 (7.46)
	Ability to return to viewing all saved information	114 (6.21)
**Self-management**		
	Checking health status and remaining up to date	58 (3.16)
	Motivating to take care of myself and contact health care services	15 (0.82)
	Preparing for the next appointment	12 (0.65)
	Supporting independence	7 (0.38)
**Helps in understanding**		
	More detailed instructions of HCPs^c^ in notes	44 (2.4)
	What was not said during the appointment	12 (0.65)
Ability to check the notes: identifying potential misunderstandings and errors		18 (0.98)

^a^A respondent may have mentioned more than one experience.

^b^EU: European Union.

^c^HCP: health care professional.

The respondents also appreciated that all health information was provided in the same place via the patient portal. Therefore, there was no need to store health information in paper format:

Compared to storing and processing prescriptions on paper, searching for them, checking their validity, and renewing them via My Kanta is a great relief in everyday life.

Respondent #4493

Many functionalities were perceived as very positive experiences. The prescription renewal was the most commonly mentioned feature (almost every third response: 566/1837, 30.81%). Other functionalities mentioned were the EU digital COVID-19 vaccination certificate; printing, uploading, and saving; acting on behalf of an adult or a child; living will and organ donation testament; SMS text message notification of prescriptions; and consent to, or denial of, the sharing of one’s own data.

The ease of use of the patient portal was reported as a very positive experience. A quarter (462/1837, 25.15%) of the respondents mentioned ease of use, meaning ease of use of the patient portal or a specific functionality or that the patient portal makes the health care process easy. Many of the respondents (368/1837, 20.03%) appreciated not only the information itself but also the quick and easy access to information:

When I was a patient in the hospital, I was able to see the lab results faster than from a doctor’s [tour]...My Kanta enables me to treat my condition myself; that is, we have agreed with the health care provider that I will monitor my laboratory results.

Respondent #0702

The respondents appreciated obtaining information at their leisure, whenever and wherever they wanted, without contacting their health care services. The information provided also supported recall, offering the possibility of returning to the care history or saved information:

You don’t have to wait in a phone line to check your information or see the results. It’s easy to monitor the child’s speech therapy records.

Respondent #0739

According to the respondents, it was important to obtain health information about themselves. They mentioned that the information provided improved self-management (eg, by helping them to keep up to date):

With the help of My Kanta, I stay up to date with where my treatment is going, in a situation where there are many visits to various examinations and outpatient clinics, both in primary health care and in specialized medical care.

Respondent #0793

The respondents also mentioned that the information provided helped them to prepare for their next appointment with HCPs:

The results of the laboratory tests were quickly visible. I had time to look at them before the doctor’s appointment and prepare questions about the results.

Respondent #0447

In addition, the respondents reported that the information provided helped them to understand what the HCP expected of them (eg, by offering more information or reminding them of earlier discussions among HCPs):

After I got home from the hospital, I didn’t get any home care instructions from the doctor. But on My Kanta, I read the doctor’s report, which also included instructions for home care.

Respondent #1171

I have found information about myself very easily, even information that was not heard and understood at the appointment.

Respondent #0675

In addition to the aforementioned points, the information provided also helped to identify potential errors and missing information.

### Patients’ Negative Experiences

Table 4 summarizes the respondents’ narratives of the very negative experiences. Similar to the reported very positive experiences, very negative experiences most frequently concerned the provision of information (the lack of information). Almost half (577/1305, 44.21%) of the respondents had a very negative experience when information was not available (Table 4). Missing information included, but was not limited to, past medical history, operations, vaccinations, appointments, diagnoses, radiology and blood test results, and information from private health care providers.

**Table 4:** Perceived very negative experiences (n=1305).

Themes		Values, n (%)^a^
**Information not available**		
	Missing information	438 (33.56)
	Delay in access	227 (17.39)
	Information from private health care is missing	31 (2.38)
	Information does not transfer to other health care system or service	21 (1.61)
	Delay in access because validation by HCP^b^ is still missing	12 (0.92)
	HCP responsible for patient does not get information	6 (0.46)
**Finding information is difficult**		
	Information difficult to find	255 (19.54)
	Disorganized system or view	50 (3.83)
	Disorganized view of prescriptions	49 (3.75)
	Multiple information levels	23 (1.76)
	Examination and test results difficult to view or compare	18 (1.38)
	Viewing and comparing HCP notes are difficult	11 (0.84)
**Specific functionality challenges**		
	Renewing prescriptions	80 (6.13)
	Acting on behalf of adult or child	59 (4.52)
	EU^c^ digital COVID-19 vaccination certificate	38 (2.91)
	Consent to, or denial of, sharing of own data	32 (2.45)
	Printing or saving	22 (1.69)
	Living will or organ donation testament	6 (0.46)
**Portal is difficult to use**		
	Challenges with logging in or out	36 (2.76)
	Issues with menu, topics, navigation, or structure	34 (2.61)
	Technical problem	27 (2.07)
	Worries about privacy	24 (1.84)
	Reading on mobile or tablet devices is difficult	21 (1.61)
	Slow	9 (0.69)
	System itself	9 (0.69)
	Need for another person’s help	6 (0.46)
	Missing or changing instructions	5 (0.38)
**Information is incorrect**		
	Incorrect notes	80 (6.13)
	Incorrect information or errors	42 (3.22)
	Errors in notes are difficult or impossible to correct	33 (2.53)
**Missing functionality**		
	Information about future appointments	63 (4.83)
	Real-time connection with health care	25 (1.92)
	Correcting or commenting on health information	15 (1.15)
	Notification of new information	12 (0.92)
	Inaccuracy of the information who viewed the shared data	10 (0.77)
	Booking an appointment	5 (0.38)
**Information difficult to understand**		
	Medical terminology difficult to understand	24 (1.84)
	Notes difficult to understand	7 (0.54)
	Wrong language (eg, Finnish instead of Swedish)	7 (0.54)
**Information is inappropriate**		
	Inappropriate notes	16 (1.23)
	HCP added information without telling the patient	10 (0.77)
	Unwanted (older) information is visible	6 (0.46)
Multiple digital channels: multiple similar systems		20 (1.53)

^a^A respondent may have mentioned more than one experience.

^b^HCP: health care professional.

^c^EU: European Union.

Furthermore, delays in access to information were frequently a very negative experience (227/1305, 17.39%). The duration of such delays ranged from days to months or even years. The respondents perceived that these delays were not always dependent on the functionality of the patient portal but rather on HCPs:

Sometimes, for example, the laboratory results appear very slowly, but the fault is not in My Kanta itself, but in the sending of the data.

Respondent #0247

A few of the respondents (11/1305, 0.84%) reported that the delay in access to laboratory test results was contingent on the HCP’s validation or acceptance. According to these respondents, it was not clear whether the test results required an HCP’s authorization:

I don’t understand why the laboratory test results taken don’t appear directly in the laboratory results, but you have to wait until the doctor has looked at them.

Respondent #3668

According to the respondents, another reason for unavailable information was the lack of integration among different systems or health care organizations, such as private health care centers or other health care centers. Not only did information seem to be unavailable, but it was also difficult to find. More than a quarter (355/1305, 27.2%) of the respondents reported difficulties in finding information, being provided disorganized information, or having difficulty viewing and comparing information.

The respondents described experiences when laboratory test results were sometimes found by clicking on examination results and, at other times, by clicking on appointments for laboratory tests. Another inconsistency was perceived in how new information was added. Information, such as laboratory test results or appointment details, was sometimes offered as a new note and, at other times, as an addition to a previous note. A few of the respondents (3/1305, 0.23%) were confused about whether the information was too extensive, such as old prescriptions or long notes about a short telephone conversation.

In addition, the respondents were uncertain about the reason for the missing information or whether there was a delay in access or whether it was simply difficult to find:

The information is difficult to find, or it is not added here.

Respondent #0829

Difficulties were perceived not only in general patient portal use but also in specific functions, such as renewing prescriptions; acting on behalf of an adult or a child; accessing the EU digital COVID-19 vaccination certificate; consenting to, or denying, sharing of their data; printing; saving; and living will and organ donation testament. General difficulties with the patient portal were described as involving challenges with logging in, navigating, correcting errors in notes, and using mobile devices. Regarding technical problems, the respondents did not see the benefits of the patient portal if it was not working:

Doesn’t work properly. You can’t always access your data. Annoying (ie, not useful).

Respondent #3655

The respondents reported difficulties with missing or changing instructions.

There were also privacy concerns:

My information was opened in a place I had not visited. I made a correction request.

Respondent #0860

Incorrect notes and information errors were mentioned, such as errors with operations, diagnoses, prescriptions, vaccinations, appointments, test results, and examinations. A few of the respondents (7/1305, 0.54%) reported that they had found other persons’ health information in their health records. Finding other persons’ information raised worries about the possibility of someone else having received their personal health information.

The respondents reported difficulties when trying to correct information. They stated that they would like to have the opportunity to comment on errors in the patient portal:

Furthermore, if the doctor has written my information incorrectly, there is no way to comment on it or request a correction.

Respondent #0677

The respondents also described functionalities that they would like to have in the patient portal. They would like to receive information about future appointments, such as the date, or about scheduled laboratory tests, so that they could prepare, for example, with questions for an HCP or if there was a need for a rapid laboratory test.

Many respondents would like to have more interactivity, such as personal contact with HCPs, booking appointments, or the possibility to comment on notes or renewed prescriptions. Notifications for new information, such as notes, new test results, and renewed prescriptions, were also mentioned:

When you’re waiting for some laboratory results, you have to go and see what’s going on many times when there’s no system where you can order a notification when the information is updated.

Respondent #3729

Additional information cannot be written in the prescription renewal request, which creates unawareness for the doctor and may lead to rejection of the prescription.

Respondent #0689

Furthermore, the respondents mentioned that they desired more specific information about the viewers (ie, HCPs) of shared health information:

For example, to whom the information has been disclosed. It could be possible to see immediately to whom, for what purpose, when, and at what time, etc.

Respondent #0157

The respondents mentioned other very negative experiences with patient portal information, such as problems understanding the information provided. Often, the medical terminology used by HCPs in the patient’s notes or other types of content in the patient portal could be difficult to understand:

The titles of the laboratory studies are difficult for the average reader to determine what they are about, for example, Research U -CtNgNhO.

Respondent #0473

The respondents reported that information can be presented in the wrong languages, such as Swedish-speaking patients receiving information in Finnish.

The respondents also reported unwanted information, such as inappropriate or unwanted older history, which was perceived as irrelevant currently, or a reminder about past negative experiences with health care:

In My Kanta, the nurse wrote inappropriate and irrelevant information. Data may not be deleted.

Respondent #4398

Although many respondents had a very negative experience with delay in access, a few of the respondents (10/1305, 0.77%) reported very negative experiences regarding accessing information that had not been discussed with an HCP beforehand or at a previous appointment, for example, laboratory test results or diagnoses:

I got the bad news before the doctor could call. Now, fortunately, information is not made visible until the doctor has called.

Respondent #4404

The respondents were also confused about having multiple similar patient portals. They reported that even HCPs had difficulties with multiple similar channels:

I don’t understand why you have to have My Kanta and Maisa. Why can’t they be only one?

Respondent #0145

### Patients’ Negative Emotions About the Patient Portal

In the free-text answers, respondents described emotions relating to their very negative experiences (83/1305, 6.36%; Table 5). The most common negative feelings described ranged from anger to frustration and anxiety (52/83, 63%). These negative feelings were associated with a specific function, usability difficulties, or other very negative experiences concerning the information provided.

**Table 5:** Perceived negative emotions relating to the patient portal (n=83).

Negative emotions	Values, n (%)^a^
Anger, annoyance, frustration, or anxiety	52 (63)
Worry about accessibility or missing bank accounts	19 (23)
Worry about one’s own or others’ (family and professionals) skills in using the portal	18 (22)

^a^A respondent may have mentioned more than 1 emotion.

Negative emotions affected users’ motivation and willingness to use the patient portal:

The system logs the user out in the middle of everything. This is frustrating because logging in is so laborious (identification, I mean). The motivation to take care of one’s affairs disappears when things are left unfinished due to being logged out.

Respondent #3795

There were also worries about individuals’ skills in using the portal, particularly about the skills of the respondents themselves, their family members, and their HCPs:

What about in the future? How will I manage when I can no longer use My Kanta or a computer, and I don’t have a loved one to take care of my affairs?

Respondent #0277

## Discussion

### Principal Findings

The patients who reported a very positive experience with the patient portal perceived better usability, and the patients who reported a very negative experience perceived worse usability of the system. Reporting a very positive experience and reporting a very negative experience explained 23% of the variation in the perceived usability score. The results suggest that even a single very positive or very negative experience is relevant when patients evaluate the usability of patient portals. The effect of reporting a very negative experience on usability was more than twice as large as that of reporting a very positive experience. Thus, patients’ experiences offer information for evaluating and improving usability from their point of view.

The open-ended questions about patients’ positive and negative experiences provided rich data about the perceived benefits and usability challenges of using the patient portal. Although usability tests are considered a gold standard in usability evaluation [51], the survey provided information about the most important usability issues from the patients’ point of view. The survey also allowed for the collection of data from a very large participant group compared with usability tests [52]. Thus, this approach for benchmarking the usability of patient portals seems to be promising regarding comparisons among different patient portals in different countries as well.

Most of the patients’ positive and negative experience narratives concerned access to the EHR, specific functionalities, and ease of use. Unavailable information was the most common very negative experience, whereas access to information was the most common very positive experience. In addition, difficulties in finding information and incorrect information were experienced negatively. Thus, very negative experiences were related to the obstacles to gaining the benefits of using the patient portal, and they were described as leading to strong negative emotions, such as anger and frustration, and the respondents who described very negative experiences provided longer responses than those who described very positive experiences. This may explain why the very negative experiences had a stronger effect on the perceived usability.

In this study, difficulties in using the patient portal seemed to be a common reason for the respondents’ very negative experiences. This large national survey study identified many kinds of usability difficulties related to specific functionalities, navigation, and privacy. The patients also identified new functionalities that were currently missing and could be useful; for example, notifications of new information or delays would help patients avoid unnecessary visits to a patient portal. Furthermore, patients would appreciate the possibility of correcting or commenting on incorrect information, and they would like to improve the patient portal by adding interactivity.

### Limitations

This research focused on 1 patient portal, My Kanta, in Finland. The survey was available for only 3 weeks after a patient logged out. Although many responses were received, the survey had a low response rate of 0.37% (4719/1,262,708). The response rate is similar to rates for earlier research in Finland (0.7%) [19] and Sweden (0.61%) [18]. However, the response rate may be underestimated because many users may have closed the portal window without seeing the survey invitation. In addition, the survey was intended for adults, but an unknown number of users were children (1.9% in 2018 [53]). Furthermore, the patient portal may be meaningful for older people who have health issues, but at the time of the survey, many healthy people logged in to the portal only to download a COVID-19 certificate or renew prescriptions. Caution is advised when generalizing the results to the whole Finnish user population, other countries, or other eHealth systems.

The survey collected self-reported patients’ experiences, and the researchers had no possibility of asking respondents specific clarifying questions. However, the survey allowed the collection of data from a large participant group, and the collected data were rich. In addition, the study’s goal was to identify patients’ subjective experiences and understand how they perceived usability.

Only 1 very positive experience and 1 very negative experience were asked of each respondent. However, the respondents had the opportunity to express themselves freely, and many described multiple experiences. Thus, reliability was improved because the respondents had the opportunity to use their own words without being guided by the researchers.

### Comparison With Prior Work

This study found positive experiences similar to the results of earlier research. Access to one’s health information was highly valued. Accessing health information via a patient portal was also one of the most common reasons for using a patient portal in earlier research [6,19,23,54-56]. Renewing prescriptions was the most frequently mentioned positive experience regarding functions. In earlier research, prescriptions were evaluated as the most useful feature [19] and one of the most commonly used features in the My Kanta patient portal [19,23,38]. Moreover, patients have been found to be most interested in using prescriptions in future eHealth services [57].

The reported benefits of using the patient portal support earlier findings, such as the convenience of patient portal access; help in remembering and understanding, for example, the care process [19,24] and what was said during the appointment [58]; and help in self-management, such as preparing for the next appointment [6,19,24,59,60].

The respondents also mentioned similar usability challenges as in earlier research, such as difficulties with navigation [16-18,38,56] and concerns with data privacy and security [23,38]. The inability to print out information was found in earlier research that used surveys that included open-ended answers [22]. Difficulties with navigation as well as data and privacy concerns were found in earlier research with methods similar to those used in this research: using a survey with open-ended questions [18,23] and using a focus group with older adults [38]. Patients with complex chronic diseases and disabilities have been found to have similar concerns related to data privacy and security as well as confusing interfaces [61]. Earlier studies also reported patients’ concerns about missing and incorrect information [19,22]. Incorrect information also increased the level of worry among older adults [38].

In this study, many of the respondents (227/4719, 4.81%) expected to receive information without delay. Similarly, in Sweden, patients expected to receive information from the national patient portal within 1 day [54], and long waiting periods were seen to potentially affect patients with cancer negatively [62]. Moreover, in the Netherlands, older adults complained of long delays in access to test results [63]. Some missing functionalities have also been mentioned in earlier research, such as the possibility of correcting errors [57,58], commenting on information [38], and notification of new information [38,58,64].

In this study, better subjective health status, older age, gender: man, and lower education were associated with higher subjective usability. Similarly, in a Norwegian study [65], older people evaluated a web-based symptom checker more positively because they were not aware of some usability problems or ignored difficult parts of the system. Future studies are needed to analyze the individual differences in evaluating usability.

### Conclusions

This survey study investigated patients’ positive and negative experiences of using the Finnish national patient portal My Kanta. First, the quantitative analysis identified an association between patients’ experiences and perceived usability. Reporting a very positive experience was related to better perceived usability, and reporting a very negative experience was related to worse perceived usability. The qualitative analysis of the experience narratives helped to identify the most important perceived benefits and usability challenges of the patient portal from the patients’ point of view.

The patients used the patient portal to obtain information for self-management and to benefit from specific functionalities, such as prescription renewal. Access to information was an essential part of the patients’ experiences. The patients used the health information provided for remembering the care history, improving understanding of the given instructions, and increasing self-management. Therefore, usability should be improved so that patients can find information efficiently, easily, and quickly. The patients in this study were frustrated about missing information and had difficulties finding information.

The results indicate that positive and negative experiences provide relevant information about perceived usability. Patients’ experience narratives can offer detailed knowledge about the benefits and challenges of improving a patient portal’s usability. The survey seems to be a valid method for collecting usability feedback and complementing usability tests with the experiences of a wider patient audience. Patient portals need further development to improve usability, patients’ experiences, possibilities to reap the benefits of patient portals, and likely impacts on patients’ health in the process.

## Appendix

Multimedia Appendix 1 The survey methodology according to the CHERRIES (Checklist for Reporting Results of Internet E-Surveys).

Multimedia Appendix 2 The survey questionnaire.

## Abbreviations

CHERRIES: Checklist for Reporting Results of Internet E-Surveys
EHR: electronic health record
EU: European Union
HCP: health care professional
SUS: System Usability Scale
UMUX: usability metric for user experience

## Data Availability

The numeric survey data will be available in the Finnish Social Science Data Archive [66].
