# Supplementary material for: Patients’ Experiences of a National Patient Portal and Its Usability: Cross-Sectional Survey Study
Source: J Med Internet Res. 2023 Jun 30;25:e45974. doi: 10.2196/45974 (PMC10365631; doi:10.2196/45974)
Supplement: Multimedia Appendix 1 [file jmir_v25i1e45974_app1.docx]

**Multimedia Appendix 1**

**The survey methodology according to the CHERRIES checklist.**

| **Item Category** | **Checklist Item** | **Description** |
| --- | --- | --- |
| Design |  |  |
|  | Survey design | The target group of the web-based survey was the national My Kanta patient portal’s logged-in patient users between January 24 and February 14, 2022. |
| Institutional Review Board |  |  |
|  | Approval | The study protocol was reviewed and approved by the Aalto University Research Ethics Committee (ethics approval number D/957/03.04/2020 Z). |
|  | Informed consent | The survey invitation included study information and the privacy notice, and answering the questionnaire was regarded as informed consent to participate. |
|  | Data protection | The survey was anonymous. The data is saved in a secured project folder at Aalto University. |
| Development and pre-testing |  |  |
|  | Development and testing | The survey was part of the NORDeHEALTH project in Finland, Estonia, Norway, and Sweden. Researchers from all 4 countries participated in the survey design. The survey was tested beforehand with 4 volunteer participants. |
| Recruitment process |  |  |
|  | Open vs closed survey | The survey invitation was sent only to the users who accessed their patient portal account between January 24 and February 14, 2022. Invitation to respond was appeared to them at log-out. |
|  | Advertising | The developer of the patient portal, the Social Insurance Institution of Finland, Kela advertised the survey on Facebook with a short animated video. The video included texts: “Have you used the Omakanta service?” and “What is your experience of using Omakanta?”, and “Your opinion is important. Let’s make the digital health services better together”. In addition, instructions for responding were given. |
| Survey administration |  |  |
|  | Web/E-mail | The online survey link was provided in a pop-up window when the person logged out the patient portal. |
|  | Context | Omakanta patient portal is provided and maintained by a government agency, the Social Insurance Institution of Finland (Kela) |
|  | Mandatory/Voluntary | It was voluntary to fill in the survey. |
|  | Incentives | No incentives were offered. |
|  | Time | From January 24 to February 14, 2022. |
|  | Adapted questioning | 24 questions were visible only for the relevant group e.g. who has read their notes, was mental health patient, was cancer patient or has found errors or something offensive on their notes. |
|  | Number of questions | 45 |
|  | Number of pages | 34 |
|  | Completeness check | None of the questions in the survey were compulsory. |
|  | Review step | Respondents were able to us a back button. |
| Response rates |  |  |
|  | Unique site visitor | The questionnaire could be accessed only by logging out from your personal patient portal account. A unique visitor was not determined. |
|  | Completion rate | 83.10% |
| Preventing multiple entries |  |  |
|  | Cookies, IP, log file analysis | None were used. |
|  | Registration | The survey invitation was sent only to the patient users who logged out from their personal patient portal account. Registration beyond that was not required, i.e. duplicate entries by respondents were not prevented. |
| Analysis |  |  |
|  | Handling of incomplete questionnaires | Only completed questionnaires were analyzed. |
|  | Questionnaires submitted with an atypical timestamp | Not measured. |
|  | Statistical correlation | No methods were used to adjust the non-representative sample. |
