# Supplementary material for: Patients’ Experiences of a National Patient Portal and Its Usability: Cross-Sectional Survey Study
Source: J Med Internet Res. 2023 Jun 30;25:e45974. doi: 10.2196/45974 (PMC10365631; doi:10.2196/45974)
Supplement: Multimedia Appendix 2 [file jmir_v25i1e45974_app2.docx]

Multimedia Appendix 2. The survey questionnaire.

Please evaluate your experience of [the system]: The system meets my needs.

1 - Strongly disagree

2

3

4

5

6

7 - Strongly agree

I don’t know

Please evaluate your experience of [the system]: The system is easy to use.

1 - Strongly disagree

2

3

4

5

6

7 - Strongly agree

I don’t know

Have you had a very positive experience with [the system]?

Yes

No

IF YES, please describe the positive experience here as clearly as possible.

Have you had a very negative experience with [the system]?

Yes

No

If YES, please describe the very negative experience here as clearly as possible.

How is your overall health?

Very good

Good

Fair

Bad

Very bad

I don’t know / I don’t want to answer

In the last 2 years, have you received care from a doctor (GP or specialist) or other health professional for [select all that apply]

Mental health condition(s)

Cancer

Other health problem(s)

No treatment

Gender

Female

Male

Other

Age

14 years old and younger

15 to 19 years old

20 to 24 years old

25 to 34 years old

35 to 44 years old

45 to 54 years old

55 to 64 years old

65 to 74 years old

75 to 84 years old

85 years old or older

Highest completed education

No formal education

Elementary school

12 years school - Upper secondary education

Higher vocational education (vocational diploma)

Higher education ≤ 3 years (first cycle -bachelor)

Higher education, >3 years (second cycle-master)

Research, (third cycle) of higher education

Do you have a health professional education?

Yes

No
